# Control of Male and Female Fertility by the *Netrin* Axon Guidance Genes

**DOI:** 10.1371/journal.pone.0072524

**Published:** 2013-08-15

**Authors:** Gunnar Newquist, Jesse Hogan, Kirsti Walker, Matthew Lamanuzzi, Micah Bowser, Thomas Kidd

**Affiliations:** Department of Biology, University of Nevada, Reno, Nevada, United States of America; Imperial College London, United Kingdom

## Abstract

The *netrin* axon guidance genes have previously been implicated in fertility in *C. elegans* and in vertebrates. Here we show that adult *Drosophila* lacking both *netrin* genes, *NetA* and *NetB*, have fertility defects in both sexes together with an inability to fly and reduced viability. *NetAB* females produce fertilized eggs at a much lower rate than wild type. Oocyte development and ovarian innervation are unaffected in *NetAB* females, and the reproductive tract appears normal. A small gene, *hog*, that resides in an intron of *NetB* does not contribute to the *NetAB* phenotype. Restoring endogenous *NetB* expression rescues egg-laying, but additional genetic manipulations, such as restoration of *netrin* midline expression and inhibition of cell death have no effect on fertility. *NetAB* males induce reduced egg-laying in wild type females and display mirror movements of their wings during courtship. Measurement of courtship parameters revealed no difference compared to wild type males. Transgenic manipulations failed to rescue male fertility and mirror movements. Additional genetic manipulations, such as removal of the *enabled* gene, a known suppressor of the *NetAB* embryonic CNS phenotype, did not improve the behavioral defects. The ability to fly was rescued by inhibition of neuronal cell death and pan-neural *NetA* expression. Based on our results we hypothesize that the adult fertility defects of *NetAB* mutants are due to ovulation defects in females and a failure to properly transfer sperm proteins in males, and are likely to involve multiple neural circuits.

## Introduction

Netrin is a diffusible laminin-like protein, characterized originally in the function of guiding axons to the source of Netrin expression [1,2]. Since discovery, the Netrins have been observed performing diverse functions ranging from neurite growth, angiogenesis and carcinogenosis to cell survival (reviewed in 3–5). Characterizing Netrin functions in different contexts has led to insights in our understanding of how Netrin and Netrin receptors function.

Fertility is a complex result of the processes of meiosis, mating, zygote formation and offspring production. The *C. elegans* Netrin homolog, *unc-6*, is required for normal egg laying function; mutants can be rescued by exogenous serotonin indicating a neural basis for the phenotype [6]. The primary defect appears to be a failure of the HSN neuron to innervate the vulval muscles, leading to a failure to expel eggs [3,7]. *unc-6* is also required for normal vulval structure including guiding the invasion of the gonad anchor cell, which leads to the formation of the vulval lumen [3,8,9]. *unc-6* is required for migration of the distal tip cell of the gonad [3,10], which could potentially affect gamete production, but this has not been demonstrated.

In mammals, hormones under central nervous system (CNS) control regulate many aspects of reproduction. Ovulation is triggered by gonadotropin releasing hormone (GnRH) via luteinizing and follicle stimulating hormones. The migration and axonal projections of the GnRH secreting neurons are disrupted in *Netrin-1* mutants [11,12]. Netrin-1 acts as a chemoattractant for migrating GnRH neurons in the chick [13], and also stimulates subsequent neurite outgrowth but may not affect neurite guidance [14]. *Netrin-1* mutants display perinatal lethality, so effects on fertility await tissue specific knockout analysis [15]. However, given the essential role of GnRH in fertility [16], and that disrupted GnRH neuron migration is thought to underlie the sex hormone defects in Kallmann Syndrome [17], it seems likely that *Netrin-1* will play a significant role in the mammalian reproductive axis.

*Netrin-1* is expressed in the follicle of mature pig ovaries and has been proposed to modulate follicular function, most likely via angiogenic effects [18,19]. However, sympathetic nerves directly innervate components of the ovary, so it is possible that Netrin-1 could be modulating neuronal signaling. Increased activity of the sympathetic nerves can promote polycystic ovary syndrome, a major cause of infertility [20,21].

We have developed a fly line that lacks both *Drosophila Netrin* genes, *NetA* and *NetB*, that survives to adulthood and can be maintained as homozygotes [22]. In the course of characterizing the adult phenotypes of *NetAB* flies, we eliminated a role for a *NetB* intronic gene, *hog*, in the *NetAB* mutant phenotype. *NetAB* flies display complex behavioral defects, and in this paper we analyze the origin of these defects with a focus on significantly reduced fertility of both sexes. Unlike *C. elegans unc-6* mutants, we see no obvious structural or connectivity defects in the reproductive tract or ovaries, suggesting a CNS origin potentially similar to the defects in mammalian *Netrin-1* mutants. *NetAB* males show statistically significant reductions in the number of eggs laid by their female partners, even when the partner is wild type. We found that the egg-laying phenotype requires *NetB*, and all adult phenotypes appear to be independent of the well-known role for *Netrins* at the CNS midline. The ability to fly can be rescued and appears to rely on neuronal survival and not positional information. The observed phenotypes may therefore be a combination of defects in the central and peripheral nervous systems as well as muscles.

## Results

### Generation of a viable *NetAB* mutant

**Figure 1:**
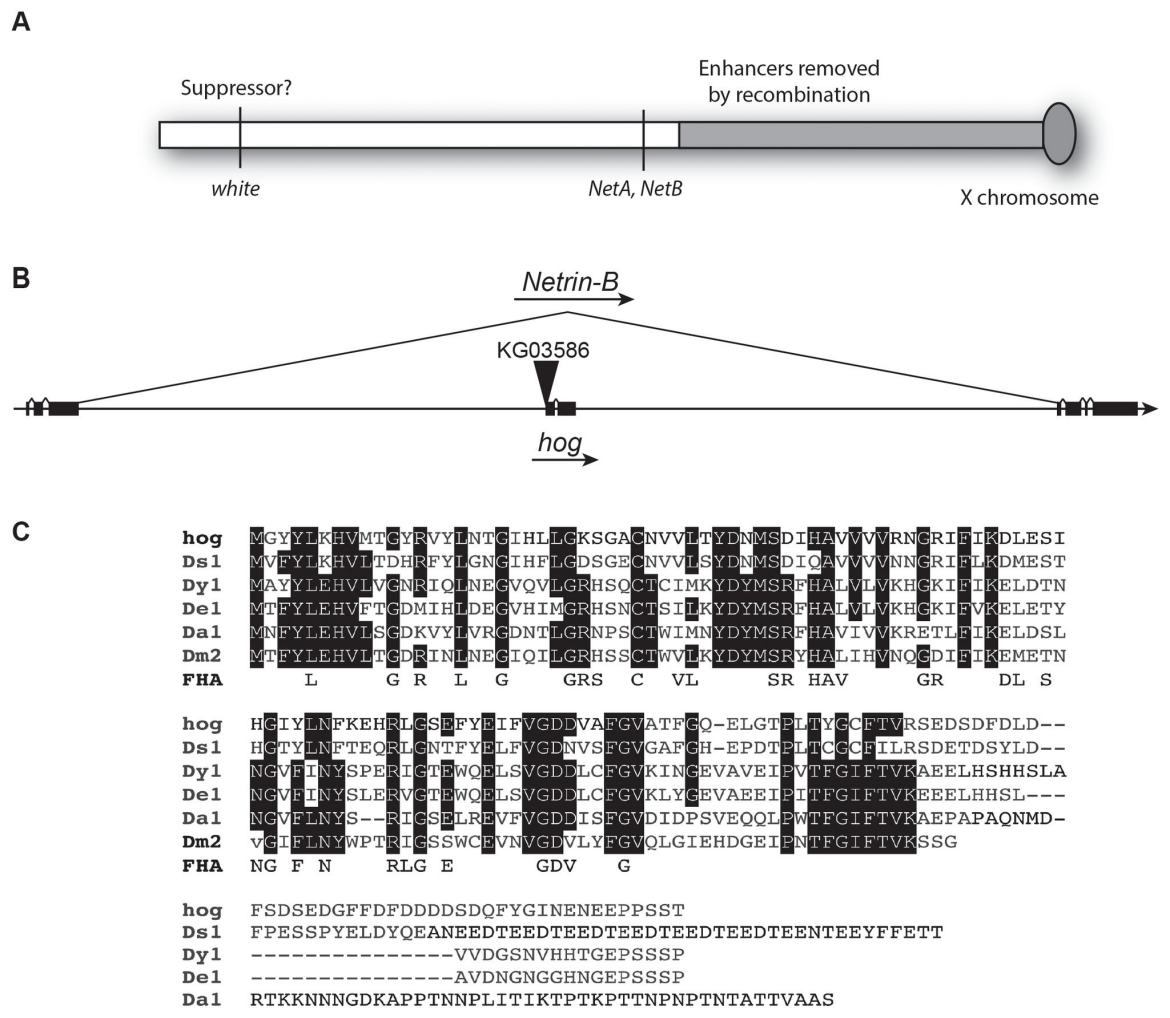
Genomic structure of the *NetAB* locus and encoded products. (**A**) Schematic view of the *NetAB* chromosome indicating approximate positions of background mutations believed to be enhancing or suppressing viability of the *NetAB* deletion. The approximate region of the chromosome exchanged by recombination is indicated by gray shading. (**B**) Diagram illustrating the location of the *hog* gene within the *NetB* gene, as well as the position of the *KG03586* transposon near the 5’ end of the hog gene. Filled boxes are exons and diagonal lines represent splicing to the mature gene products. (**C**) Predicted protein product of the *hog* gene aligned to homologous genes from other *Drosophila* species. Conserved residues are boxed. The consensus sequence for an FHA domain is shown underneath (pfam00498; cd00060). Identity of additional genes: Ds1: *Drosophila* *simulans* *GD15843*; Dy1: *Drosophila* *yakuba* *GE16495*; De1: *Drosophila* *erecta* *GG17709*; Da1: *Drosophila* *ananassae* *GF19383*; Dm2 = *Drosophila melanogaster CG34323*/*IP18436p*.

The two *Drosophila Netrin* genes are adjacent to each other on the X chromosome, most likely the product of a tandem duplication within the arthropod lineage [23,24]. Deletion of both genes is usually required to observe phenotypes, and the smallest deletion available in flies, *NetAB* ^*MB23*^, deletes only *NetA*, *NetB*, and the intervening sequence [25]. The *NetAB* ^*MB23*^ chromosome is semi-lethal as determined by the presence of the occasional hemizygous adult male. We observed that duplications for the *NetAB* region failed to rescue lethality suggesting the presence of additional mutations. Using chromosomal deletions and duplications, additional mutations were mapped to cytological intervals 13F-14B and 14F-15A. The portion of the chromosome containing these regions (proximal to *NetAB*) was replaced by recombination with a mapping chromosome (*g if f*; Figure 1A), generating an adult viable *NetAB* stock (first described in [22]).

*NetAB* mutants display thorax morphology defects, as has been observed in the Netrin receptor mutant, *frazzled* [26]. The thoraces appear split into two halves with an invagination of varying depth separating the two sides. The area of normal cuticle (darker in color with bristles present) is frequently reduced leaving plain lighter colored cuticle present. The reduction of cuticle with bristles, as well as the overall volume of the thorax is usually asymmetric and occasionally the wing is also absent. The rate of defects is 3.7% (n=373) compared to 0.32% in *OregonR* (n=619) and 0.35% in *w* ^-^ *not iso* (n=285). Flies with thorax defects were found to be entirely sterile so were excluded from all analyses except viability. Further analysis of the recombinant chromosome using chromosomal duplications suggests that there is a mutation enhancing viability of the *NetAB* stock that is tightly linked to the white locus at 3B6; the nearby *Notch* gene does not appear to be responsible as it enhances *NetAB* phenotypes (M. Alavi and T.K., unpublished observations [27]). The new stock, *NetAB* ^*∆GN*^, is referred to as *NetAB* throughout the paper for simplicity.

### Viability defects

In addition to maintaining *NetAB* as a homozygous stock, we also maintained the chromosome as a heterozygote over the FM7 balancer. We noticed that *NetAB* males appeared less often than expected; when *NetAB* males are crossed to *NetAB/FM7* females, only 35.6% of adult offspring are *NetAB* mutants whereas 50% are expected (p = 0.00016, n = 343, Chi Square test). *NetAB* mutants therefore have viability defects that we attempted to rescue through targeted *NetA* or *NetB* expression and through down-regulation of other genes. We first utilized a chromosome in which *NetA* is deleted and *NetB* has a carboxy terminal myc epitope tag added by homologous recombination (*NetA* ^*∆*^ *NetB* ^*myc*^ [25]). We found that *NetA* ^*∆*^ *NetB* ^*myc*^ rescues viability (p=0.0016, n = 162, Chi Square test, Table 1). Midline expression of both *NetA* and *NetB* has been shown to rescue the embryonic CNS phenotype [23,24], so we used the *sim-GAL4* and *rho-GAL4* drivers and *UAS-NetA* and *UAS-NetB* to test for rescue of viability. Only midline expression of *NetB* under control of the *sim* promoter rescued viability (p=0.000018, n = 256, Chi Square test, Table 1). We recently identified pan-neuronal expression of *NetB* as capable of rescuing embryonic CNS phenotypes by inhibition of apoptotic signaling [22], so we tested the pan-neuronal drivers *elav-GAL4* and *sca-GAL4* in combination with *NetA*, *NetB* and the caspase inhibitor *p35*, but saw no rescue of viability (Table 1). We additionally blocked cell death using the *H99* deletion that removes the pro-apoptotic genes *grim, rpr* and *hid* [28,29]. We also expressed *NetB* in subsets of neurons linked to fertility (see below) using *ilp7-GAL4* and *tdc2-GAL4* [30,31] without effect. Finally, we attempted to increase midline glia function by transgenic RNAi knockdown of the Pten phosphatase, a negative regulator of EGFR signaling (and also implicated in fertility [32]). EGFR signaling is critical to midline glia function [33,34], but observed no effect on overall viability (Table 1).

**Table 1:** Viability is rescued in *NetAB* mutants by midline *NetB* expression and endogenous *Netrin* expression.

**Genotype**	**Rescue?**	**p value**	**Replicates**
NetA(-)NetBmyc	***Yes***	***0.0016***	
NetAB; Y-dd4	No	0.0400	
NetAB; elav::NetB	No	0.1478	1
NetAB; ilp7::NetB	No		1
NetAB; rho::NetB	No		2
NetAB; sca::NetB	No		1
NetAB; sim::NetB	***Yes***	***0.000018***	
NetAB; tdc2::NetB	No	0.0192	
NetAB; elav::NetA	No		2
NetAB; rho::NetA	No		2
NetAB; sca::NetA	No		2
NetAB; sim::NetA	No		1
NetAB; elav::p35	No	0.3411	1
NetAB; sca::p35	No		
NetAB; sim::p35	No	0.4570	
NetAB; rho::p35	No		
NetAB; H99	No		2
NetAB; sim::ptenRNAi	No		
NetAB; rho::ptenRNAi	No		1
NetAB; elavGAL4	No		
NetAB; simGAL4	No	0.7421	

Females heterozygous for *NetAB* were crossed to stocks so as to generate F_1_ *NetAB* males of the genotype listed and the number of adult *NetAB* males that emerged was recorded. The proportion of males was compared to that of control crosses, and Chi square analysis was performed on those crosses that produced more than the expected number of *NetAB* mutants (p value for these crosses only is shown). After Bonferroni Correction, significance is set at p < 0.0023 in comparison to *NetAB* within a Chi Square Analysis. Italics and bold font denote a statistically significant difference from *NetAB*.

### Identification and analysis of *hog*, a third gene within the *NetAB* deletion

During analysis of the *NetAB* genomic locus, we noticed a small gene, *CG32595*, lying within a large intron of *NetB* (Figure 1B). We have named the gene *hog*, and the gene encodes a predicted forkhead associated domain, a binding site for phosphopeptides with a preference for phospho-threonine [35]. The *hog* mRNA pattern reflects that of *NetB*, with the addition of maternal deposition in the egg. A transposon, *KG03586*, inserted near the transcriptional start of *hog* was excised to generate two alleles lacking *hog* expression. Both alleles resulted in lack of hog mRNA, but like the parent *KG03586* insertion, *NetB* mRNA expression was unaffected. All *hog* alleles are viable. The possibility that *hog* might contribute to the adult phenotypes described in this paper led us to include *hog* alleles in our analysis.

### Egg laying defects in *NetAB* mutants

**Figure 2:**
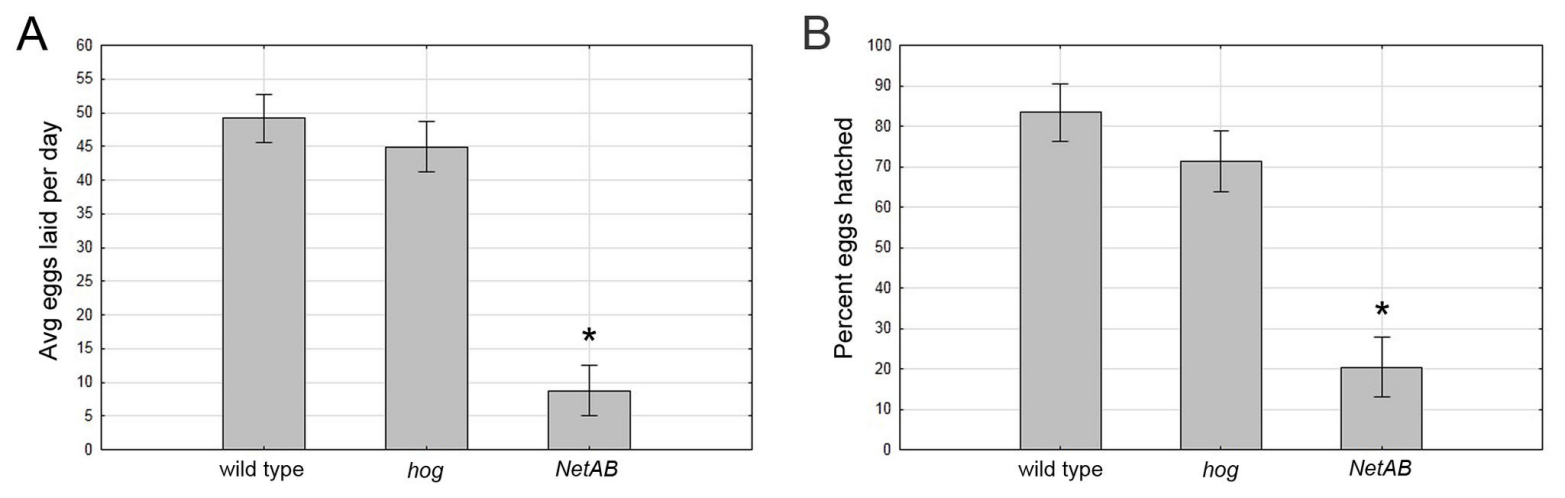
*NetAB* mutants, but not *hog* mutants, display an egg laying defect and a defect in egg viability. (**A**) Mean number of eggs laid per female averaged over 7 days with a single male of same genotype in individual chambers. Egg laying in *NetAB* is significantly reduced compared to wild type (p=0.0001, Tukey HSD within a repeated-measures ANOVA), but not in *hog* (p=0.68). (**B**) Percentage of eggs hatched after 24 hours is significantly reduced in *NetAB* compared to wild type (p=0.0001, Tukey HSD within a one-way ANOVA), but again, not in *hog* (p=0.69). Arcsine transformation was performed on percentage of eggs hatched to satisfy assumptions of analysis of variance (not shown). Data shown in bar graphs are means ± s.e.m. wild type (n=11), *hog* (n=10), *NetAB* (n=10).

The homozygous *NetAB* stock was initially quite difficult to maintain. To understand why, we examined egg laying rate and egg viability in single females mated with a *NetAB* male (Figure 2). *NetAB* mutants, but not *hog* mutants, have significant reductions in both the number of eggs laid (p=0.0001, Tukey HSD, Figure 2A), and the percentage of eggs that hatched into larvae within 24 hours (p=0.0001, Tukey HSD, Figure 2B). These results suggest that *hog* does not contribute to the fertility defects observed in *NetAB* mutants. Embryos derived from *NetAB* mothers display similar axonal phenotypes to previously characterized *NetAB* mutants suggesting some eggs fail to hatch due to neuronal defects.

### *hog*, but not *NetAB* is required for centripetal follicle cell migration

**Figure 3:**
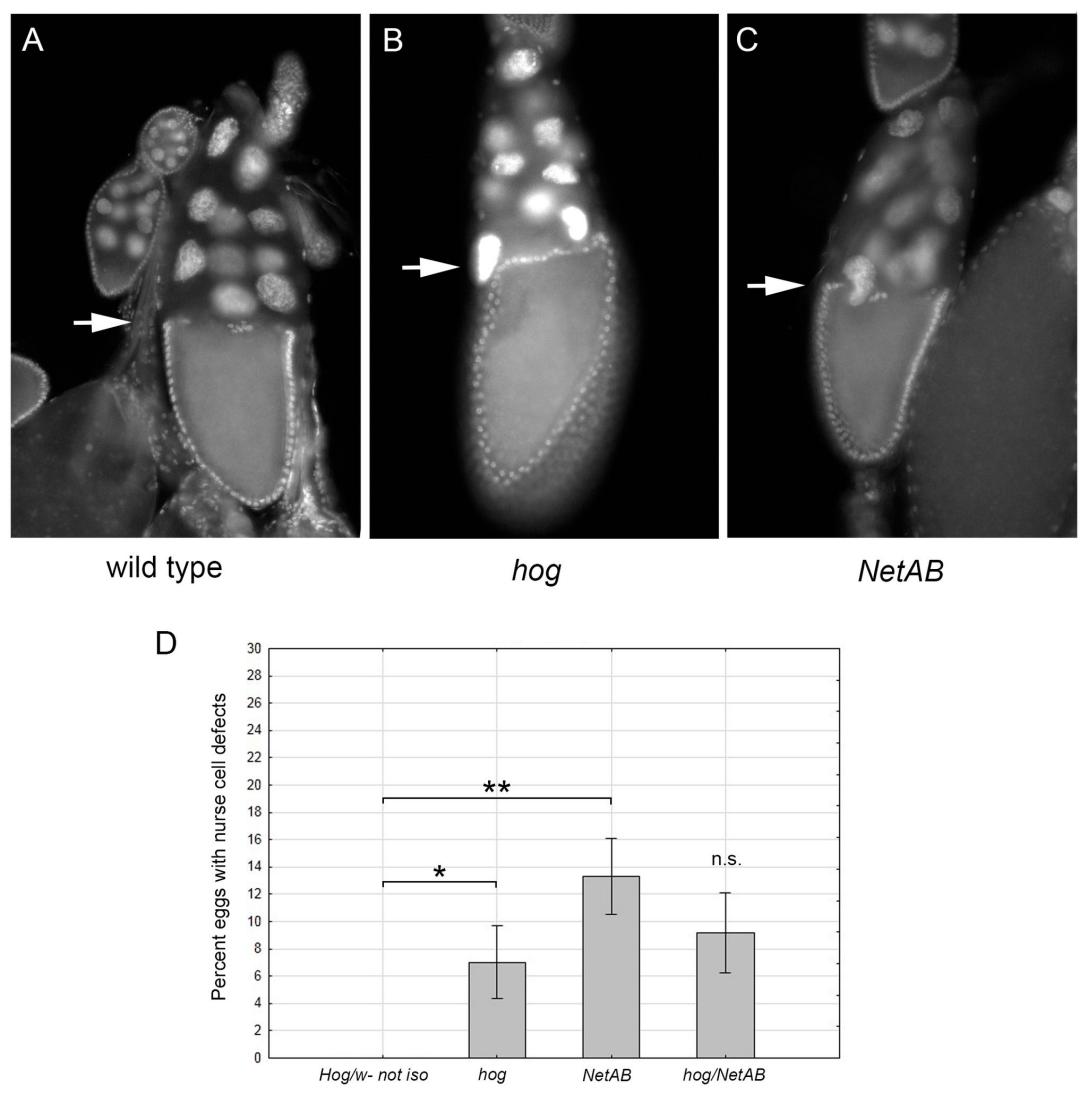
*hog* and *NetAB* mutants display nuclear positioning defects within ovaries. (**A**–**C**) Ovaries were stained with DAPI and analyzed with fluorescent microscopy to analyze nuclear position. (**A**) Wild type stage 10-11 eggs typically have a sharp boundary between the nurse cells and the developing oocyte (arrow). (**B**) *hog* mutants sometimes display nurse cell migration defects wherein a nurse cell lingers across this boundary (arrow). (**C**) *NetAB* mutants also display this nurse cell migration defect (arrow). (**D**) Mean percentage of stage 10-11 eggs that display nurse cell migration defects (*, p=0.007; **, p=0.0016; n.s. p=0.076). *Hog* heterozygotes fail to display nurse cell migration defects. Arcsine transformation was performed on percentage of eggs to satisfy assumptions of analysis of variance (not shown). Data shown in bar graph are means ± s.e.m. *hog*/wild type (n=10), *hog* (n=12), *NetAB* (n=11), *hog*/*NetAB* (n=10).

To understand the egg-laying phenotype of the *NetAB* flies, we examined oocyte development within the ovary. Oocyte development follows a stereotyped developmental program in which germline stem cells give rise to 16 cystocytes, one of which will become the oocyte [36]. The other 15 cystocytes develop into nurse cells that will deposit mRNAs and proteins into the oocyte that are required for egg development after fertilization [37,38]. At stage 10 of oogenesis somatic follicle cells migrate between the oocyte and the nurse cells, in a process termed centripetal migration [39,40]. The end result is a straight boundary between the oocyte and nurse cells (Figure 3A). Ovaries from females lacking *NetAB* or *hog* display subtle defects in the boundary with nurse cell nuclei found outside the follicle cells (Figure 3B) or protruding through the squamous follicular cells (Figure 3C). The phenotype appears to be solely due to the *hog* gene as *NetAB* mutants are statistically indistinguishable from *hog* mutants (p = 0.84, Figure 3C, D). The nurse cell phenotype has only been observed in stage 10-11 eggs in the ovary, suggesting that either this phenotype corrects itself later, or these eggs are eliminated from the population by reabsorption by the mother. It seems unlikely that this nurse cell migration defect has any consequences to the development of the egg.

### *hog* is required for normal eggshell formation

We used the 22c10 antibody to analyze ovary innervation of *NetAB* mutants (below) and noticed that a high level of non-specific background labeling frequently occurs. This labeling is restricted to the eggs and occurs in both *hog* and *NetAB* stocks giving the eggs a “hairy” appearance in the dissecting microscope (Figure S1B, C). This staining is not neuronal and resembles background expression seen when antibodies are used at too high of concentrations or when a blocking step is omitted. We counted the number of eggs within each ovary that displayed 22c10 antibody labeling and found the ectopic label was present in both *hog* and *NetAB* mutants at a much greater rate than in wild type (p=0.028, p=0.0006, respectively, Tukey HSD, Figure S1D). The number of labeled eggs was not statistically different between the *hog* and *NetAB* genotypes, so we conclude that the phenotype is specifically due to *hog*. The *Drosophila* eggshell consists of five layers formed by somatic follicle cells [41]. We hypothesize that *hog* is required for a subtle aspect of egg-shell formation that when disrupted allows ectopic binding of 22c10. A summary of *hog* defects is provided in Table 2.

**Table 2:** Summary of *hog* and *NetAB* oogenesis defects.

**Genotype**	**Eggs laid**	**Hatched**	**Nurse cell defect**	**22c10 labeled**
wild type	49.2	83.3%	0%	17.7%
*Hog*	44.9	71.3%	***7.0%***	***59.7%***
*NetAB*	***8.7***	***20.4%***	***13.3%***	***78.6%***

Column 1 lists the genotype quantified. Column 2 is the number of eggs laid by each genotype as the mean total number of eggs laid per female per day. Column 3 is the mean percentage of eggs that hatched after 24 hours. Column 4 lists the mean percentage of stage 10-11 eggs displaying a nurse cell migration defect *in ovo*. For this column, *hog* heterozygotes were used as the wild type control. Column 5 is the mean percentage of eggs labeled with 22c10 antibody *in ovo*. Italics and bold font indicate statistically significant differences from wild type.

### Morphology and innervation of the *NetAB* reproductive tract

**Figure 4:**
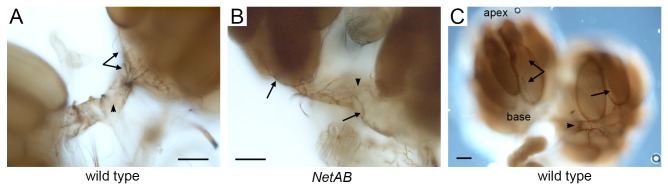
Ovaries are innervated in *NetAB* mutants. (**A**) Wild type ovary base. Each oviduct (arrowhead) and ovary is well innervated (arrows). (**B**) *NetAB* ovary base is also innervated (arrows). Arrowhead indicates one oviduct. (**C**) Wild type showing innervation toward the apex of the ovary (extra-ovarian nerves, arrows). Arrowhead indicates one oviduct. Note: The extra-ovarian innervation pattern is also present in *NetAB* mutants (not shown). Scale bars represent 10 micrometers.

As the *NetAB* fertility defects did not appear to result from disruptions to oocyte development, we assessed dissected female reproductive tracts with light microscopy and could not distinguish any differences compared to wild type. Ovarian innervation was examined using the 22c10 antibody, and despite extensive comparisons we were unable to discern any differences in the pattern between wild type and mutant (Figure 4A, B). Prior studies have used the anti-HRP antibody [42], and we found that 22c10 labels the same nerve fibers and additional nerves that travel up the outside of the ovary from the base toward the apex (Figure 4C). We named these nerves the *extra-ovarian nerves*. These extra-ovarian nerves are present in both wild type and *NetAB* mutants, and we hypothesize that they help control contractions of the smooth ovarian muscles, which also continue up toward the apex [42,43].

### Transgenic rescue of *NetAB* egg-laying defects

**Figure 5:**
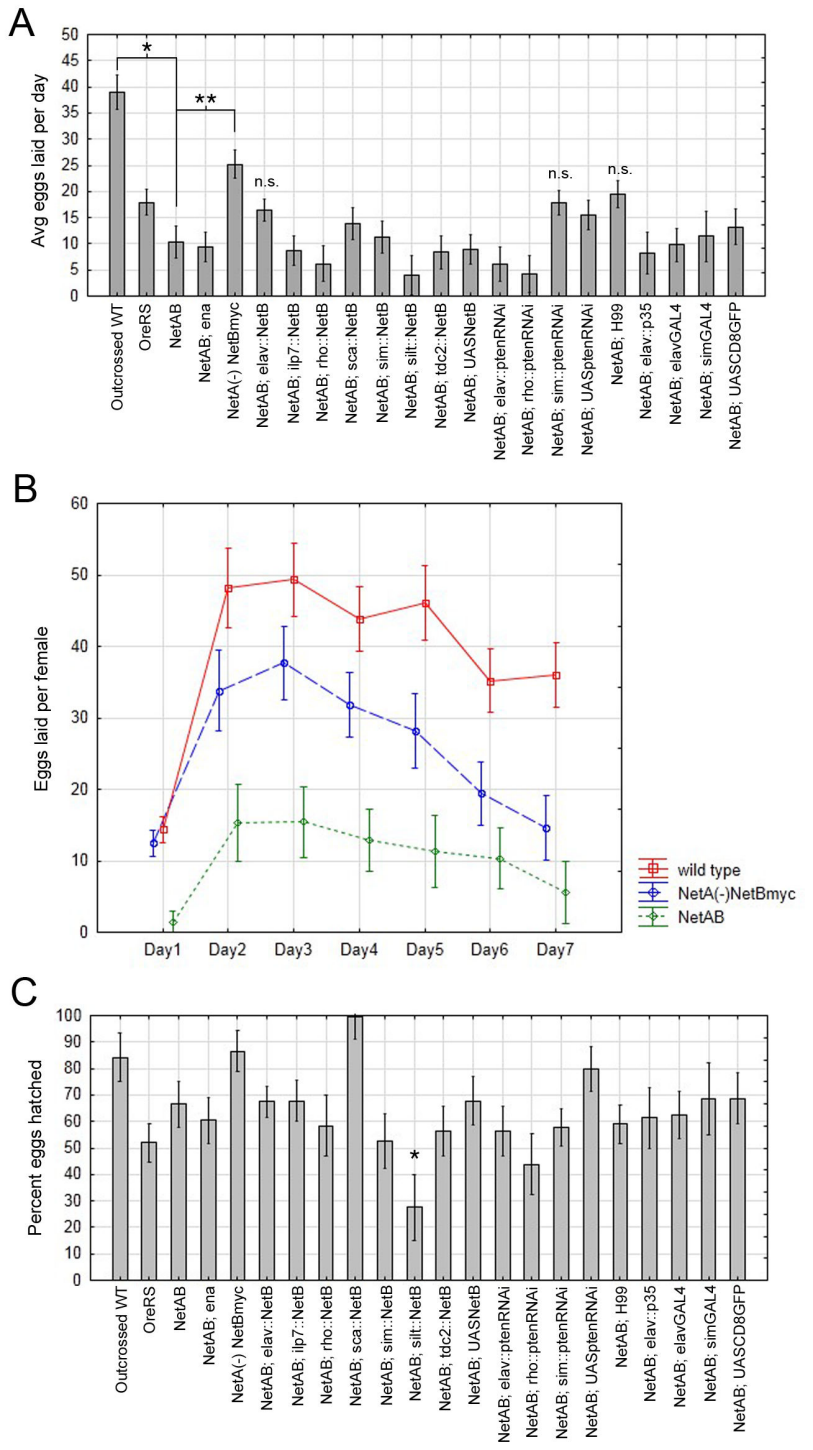
*NetAB* egg laying defect is rescued by one copy of endogenous *NetBmyc* expression but not other manipulations. (**A**) Mean eggs laid per female per day, averaged over 7 days with a wild type male. During the course of the work our wild type stock *OreRS* developed an egg-laying defect, so we used *OreRS* outcrossed to a derivative of the isogenic *w* ^-^ Exelixis stock as wild type “outcrossed WT”. *NetAB* is significantly different from wild type (p=0.00015, Tukey HSD within a repeated-measures ANOVA, *). *NetA* ^*∆*^ *NetB* ^*myc*^ rescues egg-laying (p=0.046 compared to *NetAB*, **, not significantly different from wild type, Tukey HSD within a repeated-measures ANOVA). Rescue was not observed with any other transgenic combination; all transgenic notation indicates one copy of each transgene (n ≥ 10 in all genotypes except *NetAB; slit::NetB*, n=8, *NetAB; elav::p35*, n=7, *NetAB; simGAL4*, n=5, *NetAB; rho::p35*, n=9). (**B**) Mean eggs laid per day follows similar patterns in wild type, *NetA* ^*∆*^ *NetB* ^*myc*^, and *NetAB* mutants. Number of eggs laid per female tends to be low during first 24 hrs of introduction with male and also tends to decrease by the end of 7 days of laying. Data shown in graphs are means ± s.e.m. (**C**) Percentage of eggs hatched for each of the genotypes tested. Expression of *NetB* under control of *slit-GAL4* decreases percentage of eggs fertilized (p=0.0012, Tukey HSD within a one-way ANOVA, n ≥ 10 in all genotypes except *NetAB; slit::NetB*, n=8, *NetAB; elav::p35*, n=7, *NetAB; simGAL4*, n=5, *NetAB; rho::p35*, n=9). Arcsine transformation was performed on percentage of eggs hatched to satisfy assumptions of analysis of variance (not shown). Data shown in bar graph are means ± s.e.m.

We attempted to rescue the egg-laying defect through targeted expression of *NetA* or *NetB* and downregulation of candidate genes potentially involved in *Netrin* pathways. We mated *NetAB* females carrying different transgenes to wild type males. Adding back one copy of *NetB* was sufficient to rescue *NetAB* (*NetA* ^*∆*^ *NetB* ^*myc*^, p=0.046 compared to *NetAB*, n.s. when compared to outcrossed WT, Tukey HSD, Figure 5A). *NetA* ^*∆*^ *NetB* ^*myc*^ displayed the same pattern of egg-laying over a week as wild type and *NetAB* (Figure 5B). *NetA* and *NetB* are interchangeable in most functional assays such as midline or muscle expression, but only *NetB* can promote neuronal survival when expressed in neurons [22]. We were unable to rescue egg-laying by *NetB* expression at the CNS midline, pan-neuronally or in subsets of neurons linked to fertility (*ilp7*, *Tdc2*) [30,31] or by manipulating the insulin signaling pathway with Pten RNA interference [32] (Figure 5A). We also could not rescue by removing one copy of the enabled (ena) gene, a manipulation that rescues the embryonic CNS defects of *NetAB* [44]. Our results with the *NetA* ^*∆*^ *NetB* ^*myc*^ genotype demonstrate that *Netrin* function is required in females for normal egg-laying, but our failure to rescue the phenotype by targeted *Netrin* expression suggests that the distribution of cells requiring *Netrin* is likely to be highly complex. We also examined how many eggs hatched in the transgenic phenotypes as an additional control (Figure 5C). As expected, paternal rescue (the presence of a wild type X chromosome from the father) increases the number of hatching offspring to levels statistically indistinguishable from wild type females, and only one genotype negatively affected hatching rates.

### Courtship defects

**Figure 6:**
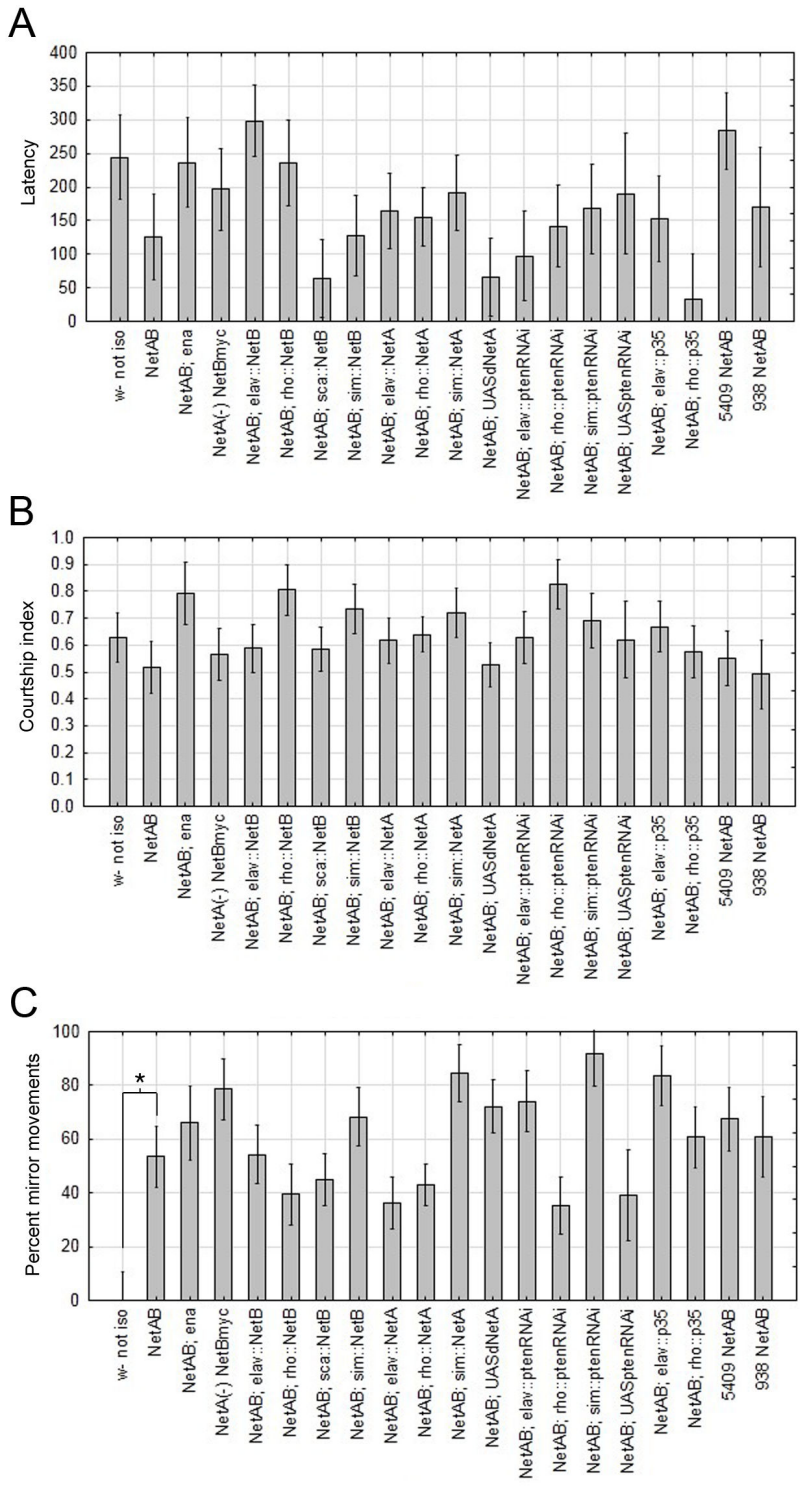
Courtship parameters are unaffected in *NetAB* mutants but male behavior is altered. (**A**) Latency to initiate courtship (the time for the first courtship specific behavior to occur) is unaffected in *NetAB* mutants. All transgenic notation indicates one copy of each transgene. Data shown in bar graph are means ± s.e.m. (**B**) Courtship index (amount of time spent by the male courting) is unaffected in *NetAB* mutants. All transgenic notation indicates one copy of each transgene. Arcsine transformation was performed to satisfy assumptions of analysis of variance (not shown). Data shown in bar graph are means ± s.e.m. (**C**) *NetAB* mutants display mirror movements during courtship behavior. This phenotype was not rescued by any manipulation attempted, including endogenous *NetBmyc* expression in the *NetAB* mutant. All transgenic notation indicates one copy of each transgene (*w-not iso* compared to *NetAB*, p=0.012, Tukey HSD within a one-way ANOVA). Arcsine transformation was performed on percentage of mirror movements to satisfy assumptions of analysis of variance (not shown). Data shown in bar graph are means ± s.e.m. n ≥ 9 in all genotypes except NetAB; UASptenRNAi, n=4 and 938 NetAB, n=5.

To further understand the *NetAB* male fertility defects, we characterized in detail several courtship parameters (Figure 6, Table 3). Wild type male *Drosophila* court females with a stereotyped series of behaviors including approaching the females, tapping with a leg, species-specific wing vibrations “singing”, tasting and attempted copulation [45–48]. Two common assessments of courtship efficacy are latency to initiate courtship and courtship index (CI), a measure of the amount of time spent courting [49]. We found that neither latency nor CI was significantly different in *NetAB* males (Figure 6A, B). However, during observation, we discovered that rather than vibrating a single wing for the female, the *NetAB* male would often vibrate both wings simultaneously. Similar “mirror movements” have been observed in *Netrin* receptor mutant humans and mice [50,51]. The number of mirror movements was found to be significantly increased in *NetAB* mutants (p=0.012, Tukey HSD, Figure 6C, Table 3), but none of the genetic manipulations tested, including those known to rescue midline axon guidance [23,24,32], were successful in rescuing this phenotype. Additionally, we found that the severity of the mirror movement defects significantly correlated with all other defects, except total number of attempts (Table 4). The embryonic CNS axon phenotype of *NetAB* is quite variable [23–25], and it would be predicted that behavioral abnormalities of individuals would vary as well. The correlation observed between different courtship defects is evidence for this hypothesis.

**Table 3:** *NetAB* males display mirror movements during courtship.

**Genotype**	**Latency**	**CI**	**Flies failing to court**	**Total attempts**	**Mirror mvt**	**Head end**	**No female**	**n**
w- not iso	244.1±63.4	0.629±.092	0%	5.3±2.5	0±10.9%	22.0±10.7%	3.0±4.5%	10
NetAB	126.1±63.4	0.517±0.097	10%	8.2±2.5	***53.5±11.5%***	17.3±11.3%	10.3±4.8%	10
NetA(-) NetBmyc	196.5±60.5	0.567±0.097	18%	5.1±2.4	***78.6±11.5%***	63.6±11.3%	4.4±4.8%	11
NetAB; ena	236.2±66.9	0.793±0.118	33%	7.4±2.7	***66.1±14.1%***	55.4±13.8%	0±5.9%	9
NetAB; elav::NetA	164.2±55.5	0.616±0.083	7%	14.6±2.2	***36.3±9.8%***	36.8±7.8%	0±4.2%	13
NetAB; sim::NetA	191.2±55.6	0.720±0.092	23%	6.8±2.2	***84.5±10.9%***	***83.4±10.7%***	13.8±4.5%	13
NetAB; rho::NetA	155.0±43.8	0.639±0.066	10%	11.0±1.7	***42.8±7.9%***	21.4±7.8%	4.4±3.3%	21
NetAB; UASdNetA	65.9±57.9	0.527±0.084	0%	15.3±2.3	***72.1±10.0%***	12.7±9.8%	3.9±4.1%	12
NetAB; elav::NetB	298.5±53.5	0.588±0.091	27%	6.9±2.1	***54.3±10.7%***	15.0±8.6%	11.9±4.6%	14
NetAB; sca::NetB	63.8±57.9	0.584±0.084	0%	16.7±2.3	***45.0±10.0%***	15.2±9.8%	1.6±4.1%	12
NetAB; sim::NetB	127.5±60.5	0.734±0.092	9%	9.4±2.4	***68.4±10.9%***	39.2±10.7%	0±4.5%	11
NetAB; rho::NetB	235.7±63.3	0.805±0.096	9%	16.8±2.5	***39.5±11.3%***	16.9±9.0%	13.3±4.8%	10
NetAB; elav::p35	152.9±63.4	0.669±0.097	10%	5.1±2.5	***83.6±11.5%***	***77.9±11.3%***	10.3±4.8%	10
NetAB; rho::p35	33.6±66.9	0.576±0.097	0%	5.0±2.7	***60.7±11.5%***	57.7±11.3%	2.5±4.8%	9
NetAB; elav::ptenRNAi	97.2±66.9	0.627±0.097	0%	11.0±2.7	***74.1±11.5%***	42.0±11.3%	0.9±4.8%	9
NetAB; sim::ptenRNAi	167.8±66.9	0.690±0.102	11%	9.8±2.7	***91.7±12.2%***	46.6±12.0%	1.0±5.1%	9
NetAB; rho::ptenRNAi	141.9±60.5	0.825±0.092	9%	15.0±2.4	***35.3±10.9%***	19.1±10.7%	4.3±4.5%	11
NetAB; UASptenRNAi	190.4±89.7	0.623±0.145	20%	13.0±4.0	***39.3±17.3%***	43.8±16.9%	1.8±7.2%	4
NetAB; 5409	283.3±57.9	0.550±0.102	33%	5.5±2.3	***67.4±12.2%***	11.7±12.0%	10.8±5.1%	12
NetAB; 938	170±89.7	0.491±0.130	0%	9.6±3.6	***60.9±15.4%***	13.0±15.2%	10.0±6.4%	5

The table lists the means of courtship parameters by genotype. Netrin mutants display deficits in symmetrical mirror movements during courtship (Mirror mvt), but not in the latency to initiate courtship (Latency), or in courtship index (CI; amount of time spent by the male courting), the total number of attempts at matings, percentage of attempts at the head end of the female, or the percentage of attempts without the female in the immediate vicinity of the male. Some transgenic manipulations resulted in an increase in these measures. Italics and bold font denote significant differences from wild type. Arcsine transformation was performed on CI, Mirror mvt, Head end, and No female to satisfy assumptions of analysis of variance (not shown). Data shown are means ± s.e.m. 938 and 5409 refer to the Bloomington Stock Center numbers for X chromosome duplications.

**Table 4:** Increase in defects in mirror movements correlates with defects in other courtship measures, except total attempts.

	**Mean**	**St. dev.**	**Latency**	**Total attempts**	**No female**	**Head end**	**Mirror mvt**	**CI**
Latency	109.0158	133.9864	1.000000	***-0.260789***	-0.055797	-0.084951	***-0.231999***	-0.072996
Total attempts	11.2158	8.3350	***-0.260789***	1.000000	-0.097480	***-0.224753***	-0.012573	0.066986
No female	0.0531	0.1443	-0.055797	-0.09748	1.000000	***0.240627***	***0.227319***	***0.173985***
Head end	0.3415	0.3384	-0.084951	***-0.224753***	***0.240627***	1.000000	***0.382957***	0.138039
Mirror mvt	0.5644	0.3866	***-0.231999***	-0.012573	***0.227319***	***0.382957***	1.000000	***0.291351***
CI	0.6383	0.2877	-0.072996	0.066986	***0.173985***	0.138039	***0.291351***	1.000000

Correlation matrix showing associations between various courtship parameters measured in all genotypes. Italics and bold font note significant correlations.

### Flight defects

The mirror movements occurring during courtship prompted us to examine the normal use of wings in *NetAB* flies. *NetAB* mutants are unable to fly, perhaps due to flight muscle innervation defects as the wings are frequently held out at an angle and droop slightly or alternatively, due to bilateral coordination problems. However, flight can be rescued by pan-neuronal expression of *NetA* (p=0.0000, Two-tailed Fischer Exact Test, Table 5) or by blocking apoptosis with pan-neural expression of the *p35* caspase inhibitor (p=0.0006, Table 5). As pan-neural *NetA* expression increases midline crossing defects in the CNS, and *NetA* has not been observed to be neurotrophic, and can actually be pro-apoptotic in some contexts [22], we are unsure how to interpret these results. It is clear that the mechanisms underlying the flight defects are fundamentally different from those involved in courtship.

**Table 5:** Flight is rescued by pan-neuronal expression of *NetA*, but not *NetB*, and by pan-neuronal expression of *p35*.

**Genotype**	**N**	**Fly**	**p value**
w-not iso	36	36	***0.0000***
NetAB	17	0	
NetAB; elav::NetA	61	40	***0.0000***
NetAB; elav::NetB	14	1	1.0000
NetAB; elav::p35	61	11	***0.0006***
NetAB; elavGAL4	37	2	0.4932
NetAB; rho::NetA	22	3	0.2326
NetAB; sca::NetA	2	2	0.3333
NetAB; sca::NetB	34	6	0.246
NetAB; UASdNetA	14	4	0.0978
NetAB; UASdNetB	38	1	1.0000
NetAB; UASp35	13	1	1.0000

Flies of each genotype were tested for their ability to fly; “n” is the number tested and “Fly” is the number that flew. After Bonferroni Correction, significance is set at p < 0.0042 in comparison to *NetAB* within a two-tailed Fischer Exact Test. Italics and bold font denote significance.

### Both sexes contribute to the *NetAB* egg laying defects

**Figure 7:**
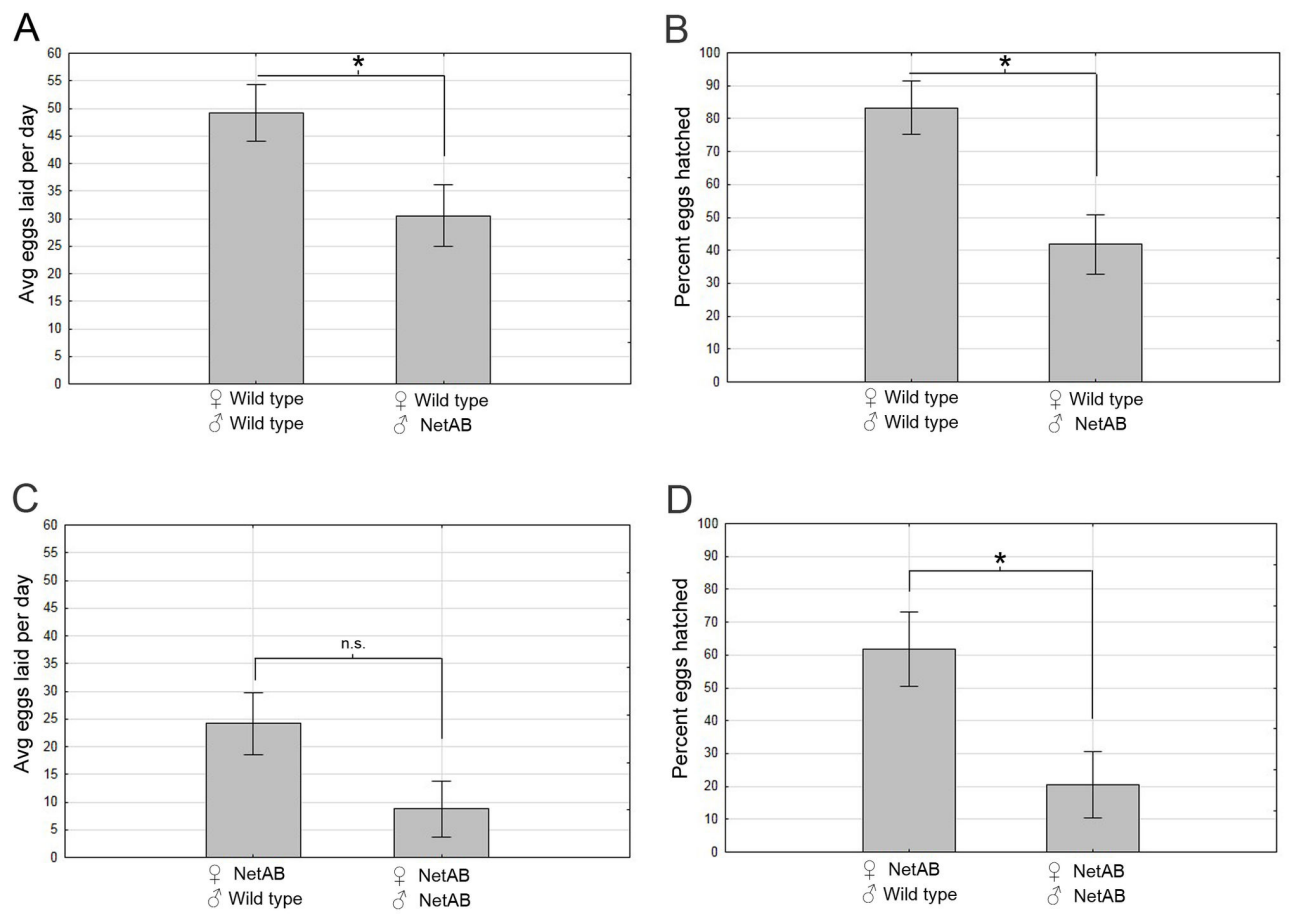
Number of eggs laid by females is decreased by the presence of *NetAB* males compared to wild type males. (**A**) Mean number of eggs laid is significantly decreased in wild type females when the male is *NetAB* (p = 0.0246, Student’s t-test). (**B**) The percentage of eggs hatched is significantly decreased when the male is *NetAB* in both wild type females (p = 0.026, Tukey HSD within a one-way ANOVA). (**C**) When the mated female is *NetAB*, the difference between wild type and *NetAB* males on the mean number of eggs laid is borderline significant (p=0.058, Student’s T-test). (**D**) The percentage of eggs hatched when the mated female is *NetAB* is still significantly reduced when the male is also *NetAB* (p = 0.030, Tukey HSD within a one-way ANOVA). Arcsine transformation was performed on percentage of eggs hatched to satisfy assumptions of analysis of variance (not shown). Data shown in bar graphs are means ± s.e.m. wild type/wild type (n=11), wild type/*NetAB* (n=9), *NetAB*/wild type (n=8), *NetAB/NetAB* (n=10).

To further understand how the *Netrins* might be affecting egg-laying ability, the role of the *NetAB* genotype in pairwise combinations with wild type flies was examined. Surprisingly, when wild type females were mated to *NetAB* males, there was a significant reduction in the number of eggs laid compared to wild type males (p=0.0246, Student’s T-test, Figure 7). Although uncoordination of the males might decrease courting success and egg laying, we did not observe differences in courtship (see below). It is therefore possible that *NetAB* male sperm composition is altered affecting fecundity [52]. *NetAB* males also had a decreased number of eggs hatching from wild type mothers (Figure 7B), even though all of the eggs should be wild type or heterozygous for *NetAB*. When *NetAB* females were mated to *NetAB* or wild type males the reduction in egg-laying observed was not quite significant (p=0.058, Student’s t-test, Figure 7C). We suspect this could be due to a floor effect (the data cannot take on lower values) as *NetAB* females frequently fail to lay eggs altogether on any given day. As expected, paternal rescue (the presence of a wild type X chromosome from the father) increases the number of eggs hatching from *NetAB* mothers (p=0.030, Tukey HSD within a one-way ANOVA, Figure 7D). If unsuccessful fertilization is the cause of the reduced egg-laying, then more eggs should be laid with time. Time course experiments during one week indicate that this is not the case as the number of eggs laid plateaus after 24 hours (Figure 5B), a pattern that resembles wild type flies albeit with greater numbers. In summary, *NetAB* females lay significantly less eggs than wild type and *NetAB* males contribute significantly to reduced egg-laying, even in wild type females.

## Discussion

This work characterizes the adult phenotypes of mutants lacking both the *Drosophila Netrin* genes. We have previously reported locomotor and negative geotaxis defects in *NetAB* mutants that can be rescued in part by inhibition of apoptotic signaling or pan-neural expression of *NetB* [22]. In this study we focus on the fertility defects of *NetAB* mutants and also describe viability and flight phenotypes. Our results indicate that the Netrins are highly pleiotropic genes affecting multiple systems as have been observed in other species [3].

We identified and exclude a role for the *hog* gene in *NetAB* phenotypes. The location of *hog* within the *NetB* gene appears to be purely the result of chance, as homologues of *hog* in other *Drosophila* species are not located within *NetB*. The non-specific binding of the 22c10 antibody to *hog* mutant eggs suggests that *hog* is required for eggshell formation, but in a very subtle manner as egg viability is unaffected.

Flying requires advanced coordination between the two sides of the body, so it is not surprising that it is affected in *NetAB* mutants. The defects observed could be due to failure to innervate flight muscles [53] or failures in interneuron connectivity in the CNS [54]. Rescue of flight by pan-neural expression of the caspase inhibitor *p35* indicates a neural origin for the phenotype. Flight was also rescued by pan-neural expression of *NetA*, which was surprising as we have previously demonstrated that pan-neural expression of *NetA increases* axon guidance errors in the embryonic CNS [22]. Nevertheless, pan-neural *NetA* expression can rescue the locomotor defects of *NetAB* flies suggesting that *NetA* expression may have a context dependent beneficial effect in a brain region controlling both behaviors such as the central complex [55,56].

*NetAB* males also display hyper-coordination of wing movements during the singing phase of courtship behavior. This behavior is strikingly similar to the mirror movements observed in human and mouse *DCC* Netrin receptor mutants [50,51]. This phenotype was resistant to rescue by all the manipulations attempted, including restoration of one copy of endogenous *NetB* and midline *NetA* expression, suggesting that the behavior relies on the localized expression of *NetA* at a non-midline location. Alternatively, the behavior may rely on both *NetA* and *NetB* in independent roles.

*NetAB* females lay fewer eggs than wild type. The origin of this phenotype does not appear to be in oocyte or ovarian development. Ovulation is regulated at multiple levels including octopaminergic neurons innervating the reproductive tract [57], secretory cells in the spermathecae and parovaria glands [58] as well as cell populations in the CNS [43,59,60]. Disruption to any one or more of these systems could be sufficient to produce the reduced egg-laying seen in *NetAB* mutants. *Drosophila* females display a post-mating switch in which increased egg-laying and decreased mating receptivity is triggered by proteins in the male semen [61]. The neuronal circuits stimulated by these proteins [62,63] could also be disrupted in *NetAB* mutants. Our transgenic manipulations focused on the classical roles for Netrins at the CNS midline as well as more recent roles as neuronal survival factors. None of these manipulations restored egg-laying, so we cannot discern whether there is a local disruption of signaling in the reproductive tract or a broader disruption of multiple cell populations (including non-neural tissues). We have occasionally observed females in which an egg has been released from each ovary simultaneously, and these eggs appear jammed at the confluence of the common oviduct. We therefore favor the hypothesis that ovulation is disrupted by the *NetAB* mutations.

*NetAB* males induce reduced egg-laying in the females they mate with. Viable offspring are produced demonstrating that mating is successful, but the normal post-mating increase in egg-laying may be diminished or absent. This suggests that transfer of sperm proteins or seminal fluid (SSFT) may be disrupted. SSFT requires a male specific neural circuit [64], and the transfer of sperm can be genetically separated from seminal protein/fluid transfer by feminizing a small subset of cholinergic neurons [65]. *Netrin* mutations may be affecting one or more of these neural subsets with a deleterious effect on SSFT.

This discussion of the phenotypes observed has focused on the classical roles of Netrins in cell migration, neural connectivity and also survival. It is quite possible that the *Netrins* could be modulating neuronal activity in the adult animal, as the Netrin receptor DCC has been shown to modulate synaptic strength in mice [66]. As noted above, altered activity in ovarian nerves is associated with infertility in humans [21].

To address the function of the *Netrins* in these fertility defects, there are many possible future directions. Promoter fragments fused to GAL4 for both *NetA* and *NetB* are now available [67,68] and testing the ability of *NetA* or *NetB* driven by these lines to rescue the defects in *NetAB* flies would likely allow the site of Netrin action to be identified. The anatomy of surrounding nerves and tissues could then be examined. Localization of the Netrin proteins in pupal and adult tissues using antibody reagents would also assist in analyzing Netrin function [69]. Functional assays of oviduct contraction would allow ovulation to be examined directly and also the role of sperm proteins to be tested [42]. Finally, the *fra* Netrin receptor can produce homozygous adults that could be tested for similar defects.

## Conclusions

The *Drosophila Netrin* genes are required in both male and female flies for optimum fertility. *NetAB* mutants also display decreased viability, a failure to fly, and males exhibit hyper-coordination of wings during courtship. All behaviors are rescued by restoring one copy of endogenous *NetB*. Flight is surprisingly restored by pan-neuronal expression of *NetA*, but not *NetB*. The control of egg-laying may reside in multiple tissues or there is a very specific requirement that is not addressed by the drivers used in this study. Future work identifying the precise positional and temporal requirements for the *Netrins* will provide insight into how the *Netrins* function in egg-laying and other adult phenotypes.

## Materials and Methods

### Genetics

The following wild type strains were used for analysis: *Oregon R* (*OreR*), *OreRS*, *w* ^-^ *not iso*, and OreRS outcrossed to *w* ^-^ *not iso* (Outcrossed WT) (*w* ^-^ *not iso* is a healthier derivative of the Exelixis isogenic *w* stock). During the course of these egg laying experiments *OreRS* developed an egg-laying defect (compare Figure 5A to Figure 2A). We therefore outcrossed *OreRS* to *w* ^-^ *not iso*, and this rescued the *OreRS* egg laying defect (“Outcrossed WT”, Figure 4A). The *NetAB* ^*MB23*^ deletion was obtained from B. Dickson and the presence of the *NetAB* deletion repeatedly confirmed by PCR, using the primers 5’ TAGCCGGTCAGTAATTTACC 3’ and 5’ TGAAGGATACGTCTACTACTGAG 3’. The *NetAB* ^*MB23*^ stock (a deletion encompassing *NetA*, *NetB*, and *hog*) was recombined with *g if f* to generate the adult viable line used in this study. Recombination resulted in a single hemizygous *Netrin* deletion mutant male that proved fertile. A stock was developed from this male by backcrossing to *NP5/FM7* females and selecting the appropriate progeny. We named the stock *NetAB* ^*∆GN*^, but refer to the stock as *NetAB* from here on for simplicity. For fertility and antibody labeling: Oregon R, *NetAB, hogB* were used. Strains for nurse cell nuclear phenotypes: *hog/w-not iso*, *hogB*, *hogB*/*NetAB*, *NetAB*. The *KG03586* transposon and transposase stocks were obtained from the Bloomington Drosophila stock center. Excision of the *KG03586* insertion was by standard techniques. Additional stocks used were obtained from the Bloomington stock Center or the labs listed in the acknowledgments. Stocks used or generated in this study: *NetAB/FM7, NetAB/FM7; UAS-dNetA, NetAB/FM7; UAS-dNetB, NetAB/FM7; UAS-ptenRNAi, NetAB/FM7; UAS-p35, NetAB/FM7; UAS-CD8-GFP, NetAB/FM7; elav-GAL4, NetAB/FM7; slit-GAL4, NetAB/FM7; sim-GAL4, NetAB/FM7; sca-GAL4, NetAB/FM7; rho-GAL4, NetAB/FM7; ilp7-GAL4, ena* ^23^ */CyOWgβ, H99/TM3, NetA* ^*Δ*^ *NetB* ^*myc*^. X chromosome duplications used (Bloomington Stock Center identification numbers): 901, 936, 938, 1527, 5409, 5594, 6251, 9144, 30235, 30236, 30237, 30260, 30261.

### Immunohistochemistry

Embryonic immunohistochemistry was performed as described in [70]. For ovarian immunohistochemistry, ovaries from each female were dissected in 1X PBS after the fertility assay had been performed. Ovaries were immediately fixed in 4% paraformaldehyde 2X PBS solution or 1:10 37% formaldehyde 1X PBS solution for 45 mins. (Note: no difference was found between fixatives.) Ovaries were rinsed with 1X PBS 0.1% Triton solution (PT) and blocked with 10% NGS PT solution for 15 mins prior to antibody label. 22c10 monoclonal antibody was applied overnight at 4 degrees C. Ovaries were again washed with PT solution and blocked for 15 mins prior to anti-mouse-HRP secondary antibody. Secondary antibody was applied overnight at 4 degrees C. After wash in PT, DAB solution was applied for 30 mins. Ovaries were immersed in 70% glycerol and analyzed under light. Only eggs visible from the position of the ovary on the slide were counted. Therefore, numbers reported represent a sample of the total egg numbers. Statistical analysis was performed using a Tukey HSD within a one-way ANOVA. Arcsine transformation was performed to satisfy the assumptions of analysis. Data shown in bar graphs are means ± s.e.m.

### Nurse cell nuclear phenotypes

Cell nucleus labeling was performed using DAPI stain on ovaries. Females of each genotype and wild type males were placed in cages together with grape agar and a small amount of yeast for three to four days from day of birth in a 25 degree C humidity-controlled environmental chamber. On the final day, ovaries from females of each genotype were dissected intact in 1X PBS. Ovaries were immediately fixed in 1:10 37% formaldehyde 1X PBS solution for 45 mins. Formaldehyde solution was rinsed with 1X PBS, and then ovaries were placed in 1X PBS with 0.1% Triton detergent. Ovaries were then mechanically disrupted to facilitate stain penetration. Ovaries were placed in DAPI 1:1000 1X PBS solution for 2 mins. Egg stage was assessed under fluorescent microscopy using the stages outlined by [71], and the total number of eggs at each stage (only stages 10-14) was recorded. Experimenters were blind to genotype during recording. Nurse cell migration defects were defined as any nurse cell crossing the boundary between the nurse cells and the developing egg at stage 10 or 11. Percentage of nurse cell defects is reported as a percentage of the total number of eggs at stage 10 and 11 in each fly. Statistical analysis was performed using a Tukey HSD within a one-way ANOVA. Arcsine transformation was performed to satisfy the assumptions of analysis. Data shown in bar graphs are means ± s.e.m.

### Viability

A reference cross of NetAB/*Y* (X) NetAB/FM7 was performed, and the number of offspring of each gender and genotype was counted. The proportion of each genotype arising from this cross was used to generate the expected genotypic values for Chi Square analysis. A statistical increase from this expected number of *NetAB* offspring was considered a rescue. If the Chi Square test resulted in significance, false positive results due to changes in the sex-ration were additionally ruled out. Each cross had a minimum of 5 flies of each gender, but the number of parentals varied. Some crosses were replicated as indicated by Tables 1 and 2. The Bonferroni Correction was applied to set statistical significance at p < 0.0023 for this assay. Flies were examined for anatomical defects of the thorax, and were scored as defective if any disruption to the normal pattern was present including central invaginations, asymmetry between the two sides of the thorax and missing wings. The number of thorax defects was recorded for wild type and *NetAB* strains.

### Behavioral Assays

*NetAB* flies with thorax defects (partial absence of thorax or deformity) were found to be entirely sterile so were excluded from all analyses except viability. All statistical analyses were performed with Statistica Software (Statsoft), or Excel (Microsoft) for Chi Square. Significance was set at *p* < 0.05. Data shown in all bar graphs are means ± s.e.m.

#### Fertility

Fertility was assessed by counting the number of eggs laid by each female in individual mating chambers for 7 days. Wild type males and females of each genotype were collected, separated on day of birth by gender, and placed in a 60% humidity-controlled incubator at 18 degrees C for 3 days on Jazz mix (sugar/yeast) to age. On the third day, each female was placed with one male in each individual mating chamber and placed on grape agar with a dab of yeast in a 25 degree incubator at 50% humidity. The grape agar/yeast was changed daily, and eggs were counted for 7 consecutive days. After eggs were counted, each agar plate was kept at 25 degrees C for another 24 hours to count how many of those hatched. For the first assay comparing wild type (OreR), *hog*, and *NetAB* females, males were of the same genotype as the females. Numbers of eggs laid and hatched under this condition were then compared to females of each genotype mated to individual wild type (OreR) males. For all transgenic analyses of egg laying, male was wild type. Data for chambers in which either member of the single pair died before the end of the 7 days was discarded. Time of collection was recorded, but not reported here. Statistical analysis was performed using a Tukey HSD within a repeated-measures ANOVA for number of eggs laid or a Student’s T-test for the comparison the contribution to number of eggs laid with wild type males and *NetAB* males. For percentage of eggs hatched, statistical analysis was performed using a Tukey HSD within a one-way ANOVA. Arcsine transformation was performed to satisfy assumptions of the analysis of variance.

#### Courtship

Courtship was assessed in hand-built, individual courtship chambers, constructed by Brian Kenton, Mechanical Engineering Department, UNR. Generally, this courtship assay was modeled after [49]. The courtship device was manufactured from two sheets of Plexiglas. Chambers were drilled into one sheet, while the other served as a trap door and transparent cover through which to video. Each chamber measured approximately 11mm in diameter and 5mm deep. 350 µL grape agar was placed in the bottom of the chamber prior to assay, limiting the vertical space to about two fly heights. One male was placed in the chamber via a mouth pipette and allowed to acclimate for 5mins. During acclimation of the male, a wild type (Oregon R) female was anesthetized under CO_2_, then decapitated and allowed to return to a standing position. The decapitated female was then placed with the male, and video recording began immediately. Each video session lasted 10 mins (600 secs) and was later analyzed for the following criteria: 1) latency to initiate courtship (defined as the time from female introduction until the first courtship attempt), 2) courtship index, (total time spent courting/(total time – latency), (CI), 3) the percentage of males that failed to initiate courtship in the 10 min trial period (no attempts), 4) number of times male attempted courtship (total attempts), 5) the percentage of total attempts where the male vibrated both wings simultaneously (mirror movements), 6) the percentage of total attempts where the male was at the head of the female (head end), and 7) the percentage of total attempts where the female was absent from the immediate vicinity (no female). Mirror movements, head end, and no female, were not mutually exclusive categories, but each could only be scored once per attempt. If a male failed to initiate courtship during the allotted 10 mins, he was given a courtship latency score of 601 secs. These males were excluded from analysis of CI, mirror movements, head end, and no female. Statistical analysis was performed using a Tukey HSD within a one-way ANOVA. Arcsine transformation was performed to satisfy assumptions of analysis of variance on CI, mirror movements, head end, and no female. A correlation matrix was performed to assess possible correlations among the measurements. 3 flies were excluded from analysis due to data entry errors. Experimenter was blind to the genotype during analysis.

#### Flight

Flying ability was assessed by dropping individual flies from their vials onto the lab bench. Each fly that escaped by flight was recorded in the affirmative for flying ability. We compared the observed number of fliers verses non-fliers of each genotype to the expected number of fliers verses non-fliers observed in *NetAB* (0 of 17) using a two-tailed Fischer Exact test. Bonferroni correction was applied to set statistical significance at *p* < 0.0042 for this assay.

## Supporting Information

Figure S1 ***NetAB* and hog mutants display ectopic antibody labeling phenotypes in the developing eggs.** (**A**) Wild type ovaries. 22c10 antibody normally labels axons of nerves, and fails to label developing eggs in the ovary. (**B**) In hog mutants, 22c10 antibody non-specifically labels inside most of the eggs, penetrating in hair-like tracts. (**C**) *NetAB* mutants display ectopic 22c10 label similar to hog mutants in developing eggs. (**D**) Mean percentage of ovarian eggs ectopically labeled. *hog* and *NetAB* mutants are significantly different from wild type (*, p=0.028, **, p=0.0006 respectively, Tukey HSD within a one-way ANOVA). Arcsine transformation was performed on percentage of eggs hatched to satisfy assumptions of analysis of variance (not shown). Data shown in bar graph are means ± s.e.m. wild type (n=11), hog (n=10), *NetAB* (n=10).
(TIF)
