# Supplementary figures and images for: Control of Male and Female Fertility by the Netrin Axon Guidance Genes
Source: PLoS One. 2013 Aug 15;8(8):e72524. doi: 10.1371/journal.pone.0072524 (PMC3744485; doi:10.1371/journal.pone.0072524)

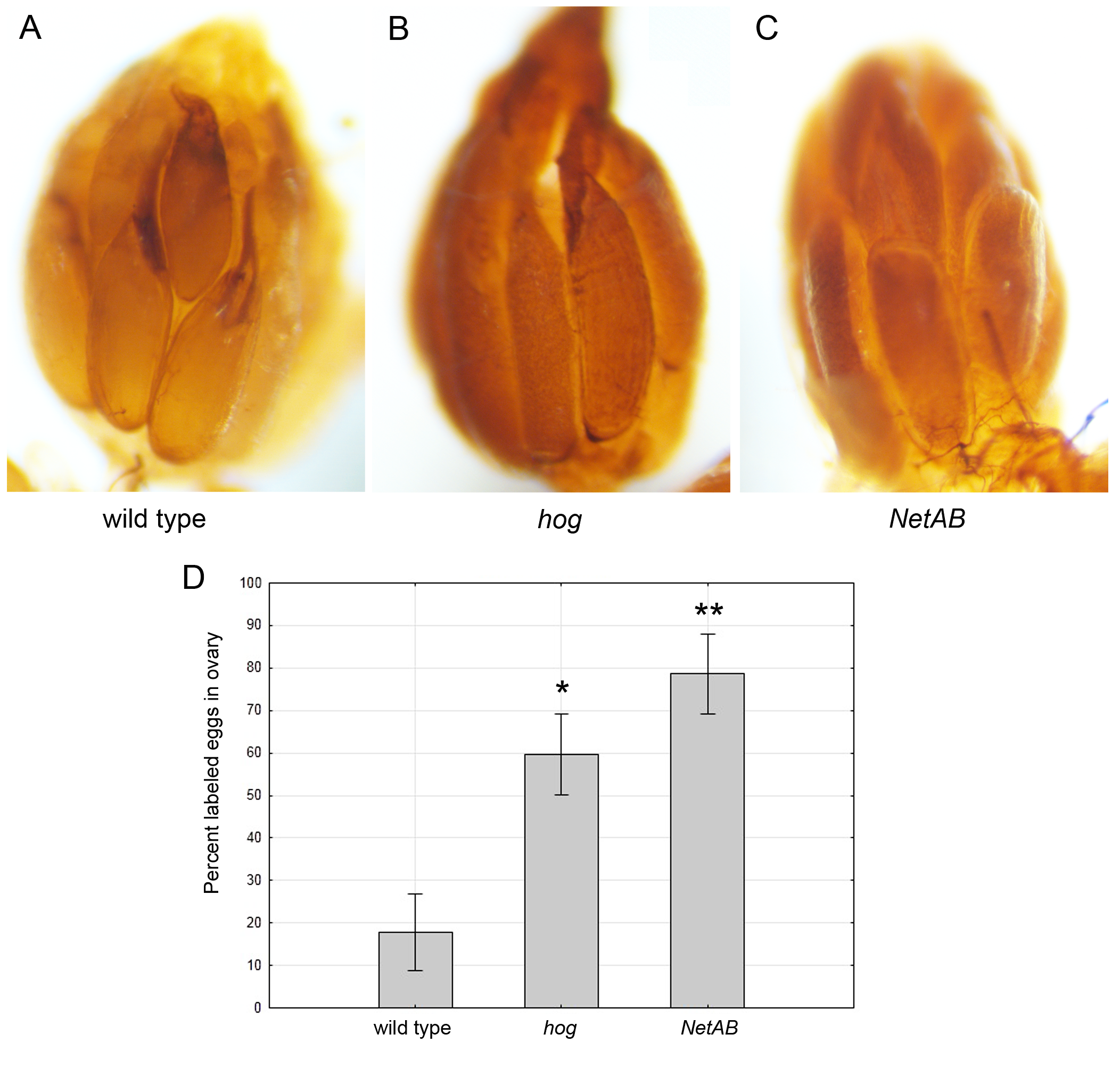

Supplement: Figure S1 — NetAB and hog mutants display ectopic antibody labeling phenotypes in the developing eggs. (A) Wild type ovaries. 22c10 antibody normally labels axons of nerves, and fails to label developing eggs in the ovary. (B) In hog mutants, 22c10 antibody non-specifically labels inside most of the eggs, penetrating in hair-like tracts. (C) NetAB mutants display ectopic 22c10 label similar to hog mutants in developing eggs. (D) Mean percentage of ovarian eggs ectopically labeled. hog and NetAB mutants are significantly different from wild type (*, p=0.028, **, p=0.0006 respectively, Tukey HSD within a one-way ANOVA). Arcsine transformation was performed on percentage of eggs hatched to satisfy assumptions of analysis of variance (not shown). Data shown in bar graph are means ± s.e.m. wild type (n=11), hog (n=10), NetAB (n=10). (TIF) [file pone.0072524.s001.tif]
